# Molecular and clinical correlates of high *FOLH1* (PSMA) RNA expression in primary and metastatic prostate cancer

**DOI:** 10.1093/oncolo/oyaf338

**Published:** 2025-10-07

**Authors:** Rana R McKay, Shayan S Nazari, Andrew Elliott, Jennifer R Ribeiro, Brent S Rose, Pedro C Barata, Deepak Kilari, Rohan Garje, Neeraj Agarwal, Norm Smith, Emmanuel S Antonarakis, Aditya Bagrodia, Himisha Beltran

**Affiliations:** Department of Medicine and Urology, University of California San Diego, La Jolla, CA 92037, United States; Medical Affairs, Caris Life Sciences, Phoenix, AZ 85040, United States; Medical Affairs, Caris Life Sciences, Phoenix, AZ 85040, United States; Medical Affairs, Caris Life Sciences, Phoenix, AZ 85040, United States; Department of Radiation Medicine and Applied Sciences, University of California, San Diego, San Diego, CA 92093, United States; Hematology and Oncology, University Hospitals Seidman Cancer Center, Cleveland, OH 44106, United States; Medical College of Wisconsin, Division of Hematology and Oncology, Milwaukee, WI 53226, United States; Genitourinary Medical Oncology, Miami Cancer Institute, Baptist Health South Florida, Miami, FL 33176, United States; Department of Medicine, Huntsman Cancer Institute (NCI-CCC), University of Utah, Salt Lake City, UT 84112, United States; Medical Affairs, Caris Life Sciences, Phoenix, AZ 85040, United States; Department of Medicine, Masonic Cancer Center, University of Minnesota, Minneapolis, MN 55455, United States; Department of Medicine and Urology, University of California San Diego, La Jolla, CA 92037, United States; Department of Medical Oncology, Dana-Farber Cancer Institute, Boston, MA 02215, United States

**Keywords:** PSMA, FOLH1, prostate cancer, lutetium-177 vipivotide tetraxetan, molecular profiling, targeted therapy

## Abstract

**Background:**

Prostate-specific membrane antigen (PSMA; *FOLH1*) is a cell-surface target for diagnostics and treatment in prostate cancer. We utilized a database of molecularly-profiled prostate tumors to evaluate clinical, genomic, and immunologic correlates of high *FOLH1* RNA expression.

**Patients and Methods:**

Prostate cancer specimens (*N* = 7,082) underwent DNA/RNA sequencing and immunohistochemistry at Caris Life Sciences. *FOLH1*-High/Low expression was defined by upper/lower quartiles or median transcripts per million. Overall survival (OS) was calculated from insurance claims.

**Results:**

Prostate adenocarcinoma had 2.97-fold higher *FOLH1* expression compared to high-grade neuroendocrine prostate cancer (*q *< 0.0001). Of 7,020 adenocarcinomas, 4,464 were primary prostate samples, 828 were lymph node metastases, and 1,686 were distant metastases. *FOLH1* expression varied across metastatic sites (highest in lymph node and lowest in liver; 1.2-fold difference, *q *< 0.05). *FOLH1*-High tumors were enriched in *AR*-V7 variants (10.1% vs 4.5%, *q *< 0.05) and associated with higher androgen receptor (AR) signaling (0.82 vs 0.78, *q *< 0.05). Conversely, *FOLH1*-Low tumors were enriched with *FOXA1* (12.2% vs 6.4%), *APC* (11.4% vs 3.0%), *PIK3CA* (5.8% vs 2.7%), and *PIK3R1* (2.6% vs 0.48%) mutations (*q *< 0.05). *FOLH1*-High tumors had a lower M1:M2 ratio, fewer Tregs and CD8+ T cells, higher immune checkpoint expression, and lower interferon signature scores. *FOLH1*-High primary tumors were more frequently microsatellite instability-High, tumor mutational burden-High, and PD-L1-positive. Among patients with metastatic tumors, median OS was improved in the *FOLH1-*High group (31.9 vs 23.3 months, *P *< .001).

**Conclusions:**

This enhanced understanding of the distinct molecular and clinical profiles of *FOLH1*-expressing prostate cancers may inform optimization of PSMA-directed treatments.

Implications for practice This comprehensive molecular analysis suggests the importance of considering tumor histology and specimen site when evaluating *FOLH1* (PSMA) expression in prostate cancer. Observed depletion of mutations in Wnt and PI3K pathway genes in *FOLH1*-High tumors may have implications for PSMA-targeted treatment response and resistance. The complex immune microenvironment associated with high *FOLH1* expression also underscores the need for comprehensive immune profiling in these tumors. The survival data presented implicates *FOLH1* as a positive prognostic indicator in prostate cancer, while outcomes of 177Lu-PSMA-617-treated patients suggest the potential benefit of patient stratification by *FOLH1* expression for treatment selection.

## Introduction

Prostate cancer is the most prevalent malignancy and the second leading cause of cancer-related deaths among men in the United States. Despite advances in diagnostic and therapeutic strategies, the disease continues to pose a significant health burden, with an estimated 313,780 new cases and 35,770 deaths projected for 2025.^1^ While the range of therapeutic treatments has expanded, offering improved survival and quality of life, the emergence of castration-resistant disease remains a critical challenge.

Prostate-specific membrane antigen (PSMA) has emerged as a crucial molecular target in the diagnosis and treatment of prostate cancer. This transmembrane glycoprotein, encoded by the *FOLH1* gene, is highly enriched in prostate cancer ­compared to healthy or benign tissue.^2–5^ While the biological function of PSMA is not well-defined, increased PSMA expression—typically detected via immunohistochemistry (IHC)—correlates with cancer aggressiveness in localized prostate cancer and has been reported as an independent predictor of poor prognosis in some^5–7^ but not all studies.^8^ Unlike PSA, a secreted biomarker, the transmembrane location of PSMA is a critical feature that has revolutionized both diagnostic imaging and therapeutic approaches.^9^ The extracellular domain of PSMA serves as an ideal target for various ligands. In diagnostics, this has led to the development of PSMA-targeted radioactive tracers, such as ^68^Ga-PSMA and ^18^F-DCFPyL, which can be visualized using positron emission tomography (PET). PSMA-PET imaging has significantly improved the detection of prostate cancer metastases, offering superior sensitivity and specificity compared to conventional imaging modalities.^10–14^ On the therapeutic front, PSMA expression in prostate cancer, particularly in individuals with metastatic castration-resistant prostate cancer (mCRPC), makes it an important target. This has spurred the development of innovative PSMA-targeted therapies, including radioligand treatment, antibody drug conjugates, and immunologic treatment strategies.^15^

Currently, lutetium-177 vipivotide tetraxetan (^177^Lu-PSMA-617) is the only regulatory-approved PSMA-targeted therapy for patients with PSMA-PET positive mCPRC, based on results of the phase III VISION trial.^16^^,^^17^ While this therapy offers another option for patients who have failed androgen receptor pathway inhibition (ARPI) and taxane-based chemotherapy, clinical responses are notably heterogeneous. This heterogeneity in response has prompted efforts to develop more precise tools to predict patient outcomes.^18–20^ Currently, these tools do not integrate molecular parameters, and optimization of patient selection for PSMA-targeted treatments remains a challenge. This gap in our understanding underscores the need for more comprehensive molecular analyses to inform patient stratification and treatment decisions.

To address this need, we utilized a database of molecularly-profiled prostate tumors to evaluate correlates of high *FOLH1* (PSMA) RNA expression. Given the distinct biological functions of PSMA and its use as a target for direct radioligand therapy, we also aimed to determine the relationship between *FOLH1* RNA expression and patient prognosis as well as treatment-related outcomes. Our findings may help refine the use of PMSA-guided therapy and uncover novel treatment opportunities for *FOLH1*-stratified subsets of patients.

## Patients and methods

### Patient specimens and ethical compliance

Formalin-fixed, paraffin-embedded specimens from 7,082 primary prostate and metastatic tumors were manually microdissected to enrich for tumor tissue and underwent molecular profiling at Caris Life Sciences, a College of American Pathologists/Clinical Laboratory Improvement Amendments-certified laboratory. This retrospective study of de-identified data was conducted in accordance with ethical guidelines (Declaration of Helsinki, Belmont Report, and U.S. Common Rule). Per 45 CFR 46.104(d)(4), this research was considered IRB exempt with a waiver of patient consent.

### Next-generation sequencing (NGS)

NGS was performed using the NextSeq (592-gene panel) or NovaSeq 6000 (whole exome with enhanced coverage of 720 clinically relevant genes) platforms (Illumina, Inc.). Variants detected were mapped to reference genome hg19 or hg38, and bioinformatic tools (SamTools, Pindel, snpFF) were incorporated to perform variant calling functions. Variants categorized as “pathogenic,” and “likely pathogenic” were counted. The threshold for TMB-High was ≥10 mutations/MB. Microsatellite instability (MSI) was measured from NGS data by counting the number of loci altered by somatic insertion or deletion and comparing to established thresholds.

### Whole transcriptome sequencing (WTS)

WTS was performed using the Illumina Novaseq 6000 platform as previously described.^21^ Raw data were demultiplexed, trimmed, counted, and aligned to human reference genome hg19 by STAR aligner.^22^ Transcripts per million (TPM) molecules were generated using the Salmon expression pipeline.^23^  *FOLH1*-High/Low groups were defined by top and bottom quartiles of *FOLH1* TPM. Androgen receptor (AR),^24^ ­neuroendocrine (NEPC),^24^ and interferon (IFN)^25^ RNA signature scores were calculated from validated gene sets. Relative abundance of immune cell infiltrates in the tumor microenvironment was calculated from WTS data using quanTIseq.^26^

### Immunohistochemistry

IHC was performed for PD-L1 using the SP142 antibody (Roche (Ventana)). Staining was scored for intensity (0, no staining; 1+, weak staining; 2+, moderate staining; 3+, strong staining) and staining percentage (0%-100%) by board-certified pathologists. The specimen was determined to be PD-L1+ if intensity was ≥2+ on ≥5% of tumor cells. Mismatch repair deficiency (dMMR) was determined by absence of staining for either MLH1 (M1), MSH2 (G2191129), MSH6 (44), or PMS2 (EPR3947). In case of discordance between MSI status by NGS and dMMR status by IHC, IHC results were prioritized.

### Statistical analysis

Kruskal–Wallis, chi-square, and Fisher’s exact tests were used to determine significance between groups. Adjusted *P*-value (*q*-value; Benjamini–Hochberg procedure) <.05 was considered statistically significant. Spearman Rank was used to determine correlations. Analyses were conducted in GraphPad Prism 10.4.1 and Python 3.12.

### Real-world clinical outcomes

Survival information was inferred from insurance claims. OS was calculated from tissue collection to last contact, and time-on-treatment was calculated from start to end of treatment. Kaplan–Meier estimates were calculated for *FOLH1*-High and *FOLH1*-Low patient groups, which were stratified by median *FOLH1* expression so as to retain sufficient patient numbers for analysis. Statistical testing was performed using log-rank test, with *P *< .05 considered significant.

## Results

### Patient cohort

**Figure 1.**
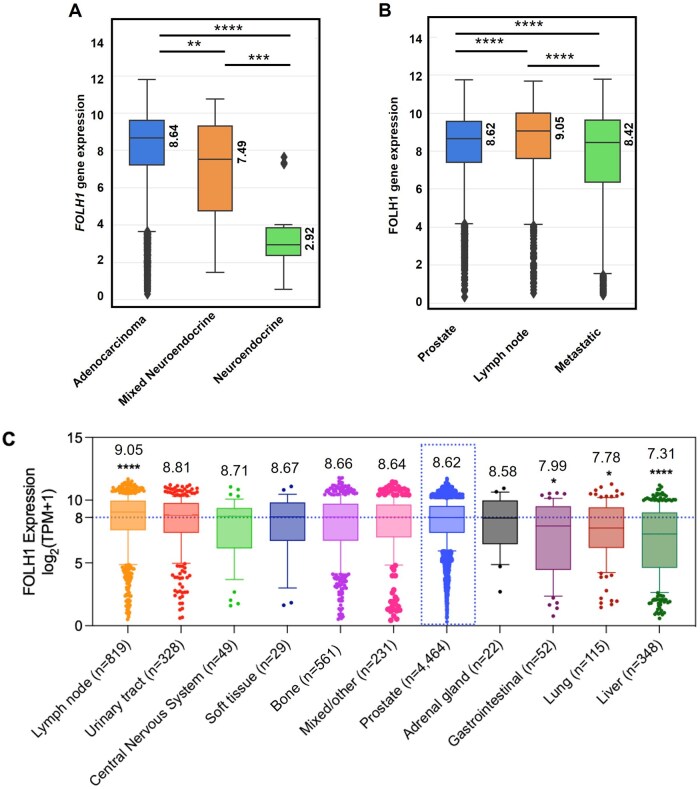
*FOLH1* Gene Expression in Prostate Cancer. (A) Distribution of *FOLH1* expression across prostate cancer histological subtypes. *FOLH1* gene expression is represented as log_2_ transcripts per million (TPM) for adenocarcinoma (*n* = 7,020), mixed neuroendocrine tumors (*n* = 40), and neuroendocrine tumors (*n* = 22). **q < 0.01; ***q < 0.001; ****q < 0.0001. (B) Comparison of *FOLH1* expression [log_2_ (TPM + 1)] between primary prostate (*n* = 4,464) compared to lymph node metastases (*n* = 828) and other metastatic prostate samples (*n* = 1,686) in cases of prostatic adenocarcinoma within the Caris cohort. The middle line indicates the median. ****q < 0.0001. (C) Median *FOLH1* gene expression [(log_2_ (TPM + 1)] in primary and metastatic sites within the Caris cohort. The dotted horizontal line marks the median *FOLH1* expression in primary prostate samples, illustrating the relative expression in metastatic sites. *q < 0.05; ****q < 0.0001.

According to histological assessment, 7,020 of 7,082 analyzed prostate specimens were labeled adenocarcinoma, 40 were mixed adenocarcinoma with poorly differentiated neuroendocrine carcinoma, and 22 were poorly differentiated neuroendocrine carcinoma including pure small-cell carcinomas. *FOLH1* expression was significantly lower in the neuroendocrine (2.92 log_2_[TPM + 1] (*q *< 0.0001) and mixed adenocarcinoma/neuroendocrine specimens (7.49 log_2_[TPM + 1]) (*q *= 0.008) compared to adenocarcinoma (8.64 log_2_[TPM + 1]) (Figure 1A). We therefore proceeded with analysis of the adenocarcinoma specimens only. The adenocarcinoma cohort comprised 4,464 (63.5%) primary prostate, 828 (11.8%) lymph node metastases, and 1,686 (24.0%) distant metastases, including bone (8.0%; *n *= 561), liver (4.9%; *n *= 348), and lung (1.6% *n *= 115), among others. The median age of all patients was 68 years (35->89), and patients with metastatic tumors were significantly older (70 years [39->89]) compared to patients with primary prostate tumors (*q *< 0.0001) (Table 1).

**Table 1.** Clinical and demographic characteristics of the study cohort.

Prostatic adenocarcinoma	Total (*N*)	FOLH1_Q1 (%)	FOLH1_Q2 (%)	FOLH1_Q3 (%)	FOLH1_Q4 (%)	*q*-value
**Specimen sites**	7020^a^	1721 (24.5)	1763 (25.1)	1766 (25.1)	1766 (25.1)	N/A
**Prostate**	4464	980 (21.9)	1251 (28.0)	1194 (26.7)	1039 (23.2)	N/A
**Lymph node**	828	180 (21.7)	155 (18.7)	196 (23.7)	297 (35.8)	N/A
**Distant metastatic^b^**	1686	548 (32.5)	350 (20.8)	366 (21.7)	422 (25.0)	N/A
**Median age, *N* [range]**	68 [35->89]	69 [35->89]	67 [36->89]	68 [39->89]	68 [35->89]	0.0002
**Median age, *N* [range] ­(metastatic sites)**	70 [39->89]^c^	71 [45->89]	70 [44->89]	71 [39->89]	71 [47->89]	0.712

a Includes 42 samples with unclear specimen site.

b Distant metastatic sites are shown in Figure 1c.

c Compared to patients with specimens collected from prostate sites, patients with metastatic tissue collection were significantly older (*q *< 0.00001).

We observed elevated *FOLH1* gene expression in lymph node metastases (9.05 log_2_[TPM + 1]) *q *< 0.0001) compared to primary prostate (8.62 log_2_[TPM + 1], or other metastatic sites (8.42 log_2_[TPM + 1], *q *< 0.0001) (Figure 1B). Conversely, *FOLH1* expression was significantly lower in gastrointestinal (GI) (7.99 log_2_[TPM + 1], *q *= 0.048), lung (7.78 log_2_[TPM + 1], *q *< 0.016), and liver metastases (7.31 log_2_[TPM + 1], *q *< 0.0001) compared to prostate (Figure 1C). Lymph node metastases had proportionally greater numbers of *FOLH1*-High tumors (35.8%), while other metastatic sites displayed a higher proportion of *FOLH1*-Low tumors (32.5%) (Table 1). For all subsequent molecular analyses, we stratified subgroups of prostate adenocarcinoma specimens (primary tumors, lymph node metastases, and other metastatic sites) into *FOLH1*-High and *FOLH1*-Low according to top (Q4) and bottom (Q1) quartiles of gene expression. Tumor sites included in “other metastatic sites” are shown in Figure 1C.

### Co-occurring genomic alterations in FOLH1 groups

**Figure 2.**
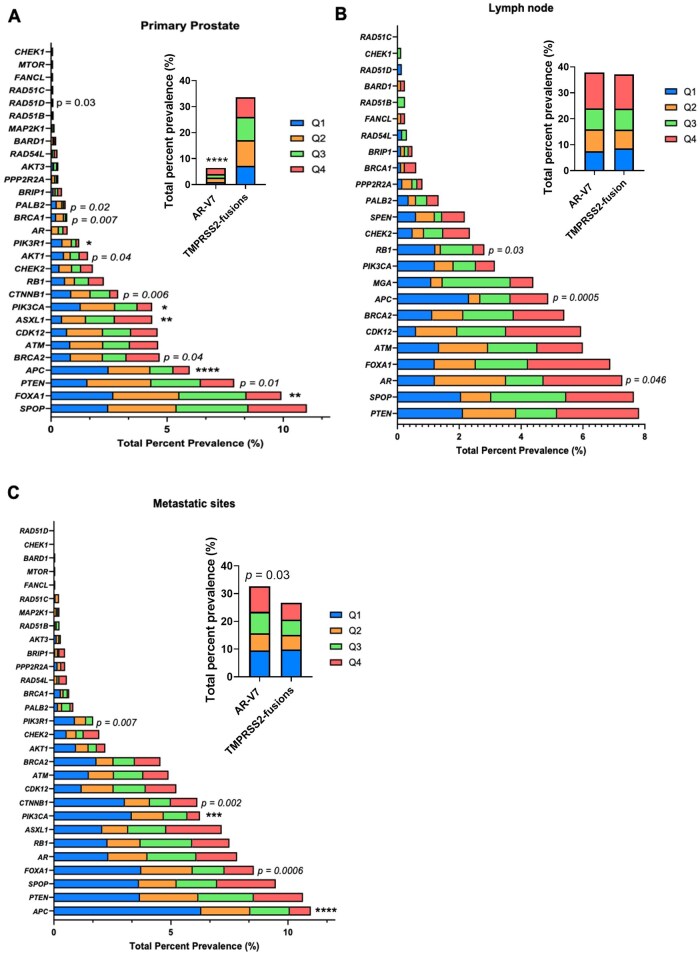
*FOLH1*-associated genomic landscape. Next-generation sequencing (NGS) was used to identify pathogenic/likely pathogenic mutations, while fusions were identified from whole transcriptome sequencing (WTS) data. Prostate samples were stratified by quartiles of FOLH1 gene expression (Q1 = low; Q4 = high). The analysis was performed in primary prostate samples (A), lymph node metastases (B), and all other metastatic sites (C). Significance was determined using chi-square or Fisher’s exact tests. *q < 0.05; **q < 0.01; ***q < 0.001; ****q < 0.0001. *q*-values indicate an adjusted significant difference, while trends are indicated by associated non-adjusted *P*-values.

NGS analysis revealed several genes with significantly different alteration rates between *FOLH1*-High and *FOLH1*-Low groups by specimen site (Figure 2). In primary prostate sites, *AR*-V7 splice site variants were more frequent in *FOLH1*-High tumors (10.1% vs 4.5%, *q *< 0.0001). *ASXL1* mutations were also enriched in *FOLH1*-High tumors (6.9% vs 2.0%, *q *< 0.01), although it is possible these are clonal hematopoiesis-derived variants detected in tissue or a passenger effect of dMMR and/or MSI-High.^27^^,^^28^ Notably, we observed depletion of mutations in Wnt and PI3K/AKT/mTOR pathways in *FOLH1*-High tumors compared to *FOLH1*-Low. Specifically, *FOXA1* (10.8% vs 12.2%, *q *< 0.001), *APC* (3.0% vs 11.6%, *q *< 0.0001), *PIK3CA* (2.7% vs 5.8%, *q *< 0.040), and *PIK3R1* (0.48% vs 2.3%, *q *< 0.040) mutations were significantly depleted in *FOLH1*-High tumors. We also observed a non-statistical trend toward fewer mutations in *PTEN*, *BRCA1/2*, *CTNNB1*, *AKT1*, *PALB2*, and *RAD51D* in *FOLH1*-High tumors (*P *< .05) (Figure 2A).

Examining lymph node metastases, the *FOLH1*-High group displayed a non-statistical trend toward more *AR* mutations and fewer *APC* and *RB1* mutations (*P *< .05) (Figure 2B). In *FOLH1*-High tumors from other metastatic sites, *APC* (*q *= 4.32e-12) and *PIK3CA* (*q *= 0.0004) mutations were significantly reduced, and there was a non-statistical trend toward increased *AR*-V7 variants and decreased *FOXA1*, *CTNNB1*, and *PIK3R1* mutations (*P *< .05) (Figure 2C).

Collectively, these data illustrate an association of high *FOLH1* expression primarily with depletion of mutations across specimen sites, particularly in genes involving the Wnt and PI3K/AKT/mTOR pathways. However, there were also some differences between *FOLH1*-High and *FOLH1*-Low groups that were unique to each specimen site.

### FOLH1 expression correlates with AR signaling

**Figure 3.**
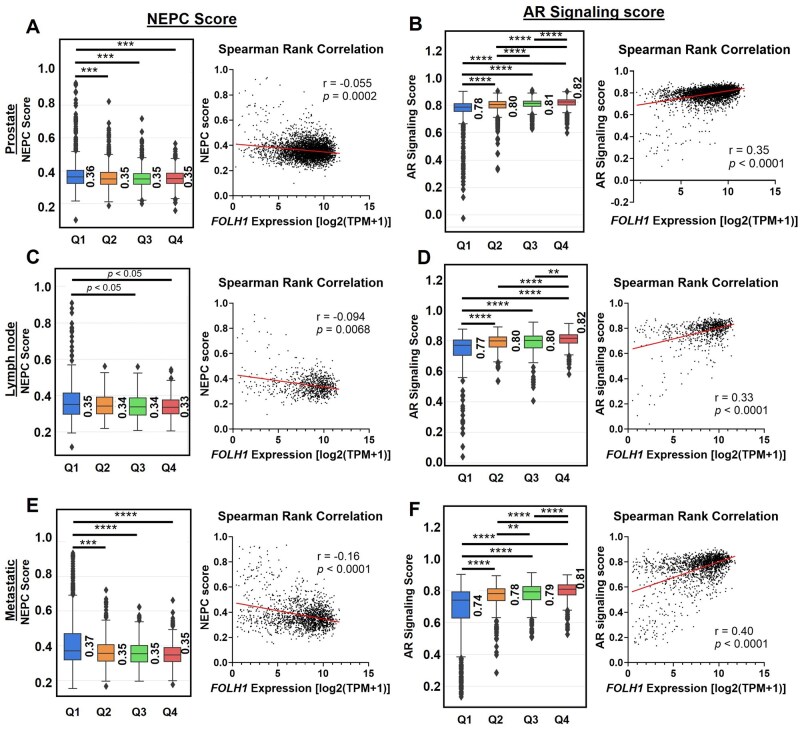
Association of *FOLH1* expression with androgen receptor (AR) signaling and neuroendocrine prostate cancer (NEPC) signature scores. Spearman rank correlation of *FOLH1* gene expression (TPM) and AR signaling scores or NEPC scores in primary prostate (A-B), lymph node (C-D), other metastatic sites (E-F). Insets show median AR signaling and NEPC scores in *FOLH1-*High and *FOLH1*-Low groups. **q < 0.01; ***q < 0.001; ****q < 0.0001. TPM = transcripts per million.

Poorly differentiated neuroendocrine prostate cancer (NEPC) is thought to evolve through clonal selection and lineage plasticity in response to AR-targeted therapy but can also occur “de novo”.^24^^,^^29^ Small-cell carcinoma is the most common histology within NEPC and associated with low or absent AR and low PSA and other AR-target genes. Even within adenocarcinoma samples, as we have analyzed here, neuroendocrine features may be present and detected using a NEPC gene expression signature score.^24^ Examining the association of *FOLH1* with signatures corresponding with AR signaling versus NEPC revealed a positive correlation of *FOLH1* with AR signaling scores and a modestly negative correlation with NEPC scores, highlighting the inverse relationship of these two signatures and the need for non-PSMA-targeted approaches for prostate tumors with neuroendocrine features. Similar results were observed in prostate, lymph node, and distant metastatic sites (Figure 3).

### FOLH1-High is associated with an immunosuppressive tumor microenvironment

**Figure 4.**
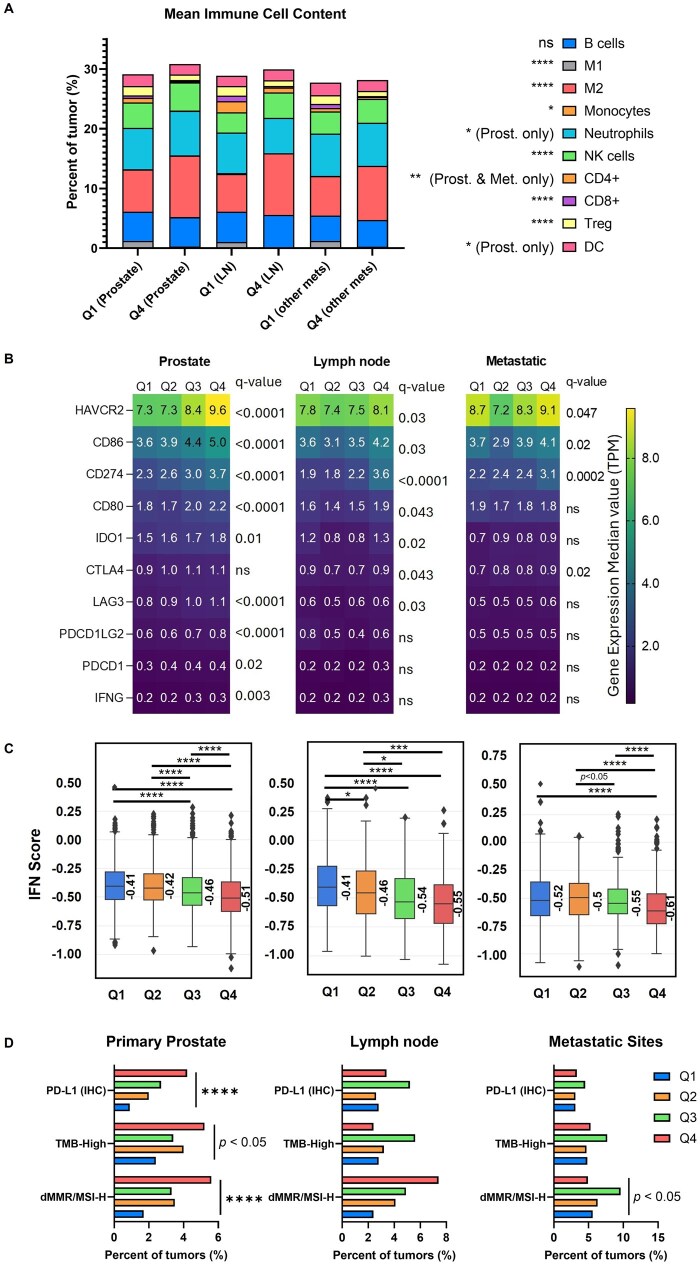
*FOLH1*-associated tumor microenvironment. (A) QuanTIseq computational immune cell infiltration in *FOLH1-*Low (Q1) and *FOLH1-*High (Q4) in primary and metastatic sites. LN = lymph node. (B) Immune checkpoint gene expression according to *FOLH1* expression quartiles (Q1, Q2, Q3, and Q4) in primary prostate, lymph node metastases, and other metastatic sites. (C) Interferon (IFN) scores according to *FOLH1* expression quartiles. (D) Percentage of tumors that were dMMR/MSI-High, TMB-High, and PD-L1(+) in *FOLH1-*Low (Q1) and *FOLH1-*High (Q4) in primary prostate, lymph node metastases, and other metastatic sites. *q < 0.05; **q < 0.01; ***q < 0.001; ****q < 0.0001. *q*-values indicate an adjusted significant difference, while trends are indicated by associated non-adjusted *P*-values.

Investigating the tumor immune microenvironment by quanTIseq,^26^ significant differences (*q *< 0.05) were noted for most immune cell subsets between *FOLH1*-High and *FOLH1*-Low groups (Figure 4A). At all tumor sites, the enrichment of M2 macrophages and depletion of M1 macrophages in *FOLH1*-High tumors was most striking, resulting in a lower M1:M2 ratio suggestive of immunosuppression (*q *< 0.0001) (Figure 4A). T regulatory cells (Tregs) and CD8+ T cells were also significantly depleted in *FOLH1*-High primary prostate tumors, lymph node metastases, and other metastatic tumors, while natural killer cells were slightly elevated (*q *< 0.0001) (Figure 4A).

Transcriptomic analysis revealed significantly higher immune checkpoint gene expression in *FOLH1-*High compared to *FOLH1*-Low tumors, which was observed for *HAVCR2* (TIM-3) (9.6 vs 7.3, *q *< 0.0001), *CD86* (5.0 vs 3.6, *q *< 0.0001), *CD274* (PD-L1) (3.7 vs 2.3, *q *< 0.0001), *CD80* (2.2 vs 1.8, *q *< 0.0001), *IDO1* (1.8 vs 1.5, *q *= 0.01), *LAG3* (1.1 vs 0.8, *q *< 0.0001), *PDCD1LG2* (PD-L2) (0.8 vs 0.6, *q *< 0.001), *PDCD1* (PD-1) (0.4 vs 0.3, *q *= 0.02), and interferon (IFN)-γ (0.3 vs 0.2, *q *= 0.003) **(**Figure 4B**).** While IFN-γ gene expression was increased, there was an overall significant decrease in the IFN signature score in *FOLH1*-High tumors (*q *< 0.0001) (Figure 4C), providing further evidence of a more immunosuppressed microenvironment.

We next considered immuno-oncology biomarkers, including TMB (≥10 mutations/MB), PD-L1 (measured by IHC using the SP142 antibody with a positive threshold of ≥2+ intensity on ≥5% of tumor cells), and dMMR/MSI-High (measured by IHC and NGS, respectively). There was a greater percentage of dMMR/MSI-High tumors (5.6% vs 1.7%, *q *< 0.0001), TMB-High tumors (5.2% vs 2.4%, *P *= .011; *q *= 0.251), and PD-L1(+) tumors (4.2% vs 0.9%, *q *< 0.0001) in *FOLH1*-High compared to *FOLH1*-Low primary tumors, but there were no significant differences in lymph node or other metastatic sites. Rates of these markers were generally low (<10%), regardless of *FOLH1* expression (Figure 4D).

### Survival and treatment outcomes

**Figure 5.**
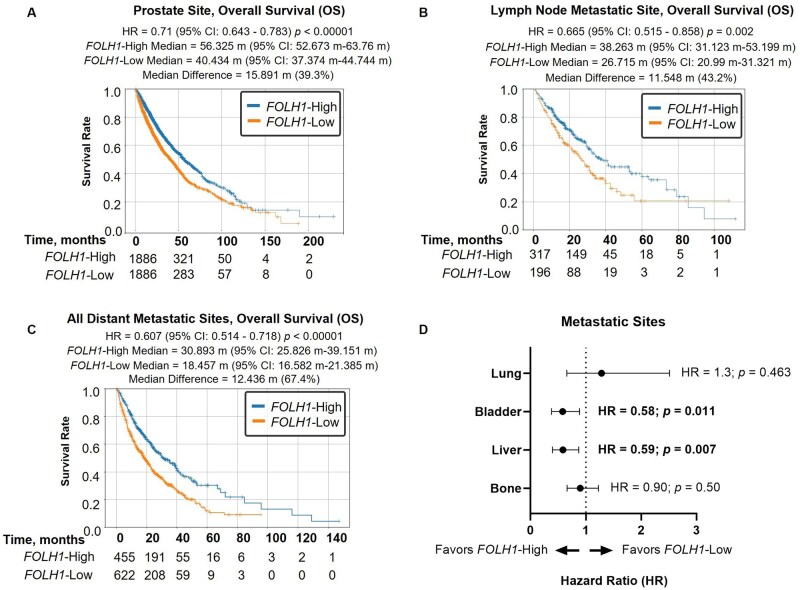
Prognosis associated with *FOLH1* expression in prostate cancer. (A-C) Kaplan–Meier curves show overall survival (OS), calculated from collection to last contact, in *FOLH1*-High and *FOLH1*-Low groups among all prostate cancer specimens (A), lymph node metastatic site (B), and all other metastatic sites (C). (D) Forest plot shows hazard ratios (HR) with 95% confidence intervals (whiskers) comparing *FOLH1*-High to *FOLH1*-Low groups among patients with bone, liver, bladder, or lung metastases. *FOLH1* groups were stratified by median gene expression.

**Figure 6.**
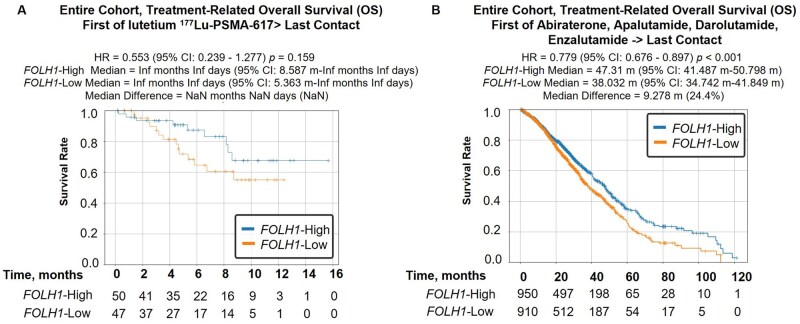
Treatment outcomes associated with *FOLH1* expression in prostate cancer. (A) Kaplan-Meier curve shows overall survival (OS), calculated from start of treatment with lutetium Lu 177 vipivotide tetraxetan until last contact, in *FOLH1*-High and *FOLH1*-Low groups of patients with prostate cancer. (B) Kaplan–Meier curve shows OS, calculated from start of treatment with AR pathway inhibitor (ARPI) (abiraterone, apalutamide, enzalutamide, or darolutamide) until last contact in *FOLH1*-High and *FOLH1*-Low groups, among all prostate cancer specimens. *FOLH1* gene expression levels were stratified by quartiles 4 vs 1 (Q1: *FOLH1*-Low, Q4: *FOLH1*-High).

When comparing OS of patients stratified by *FOLH1* expression, patients with *FOLH1*-High tumors displayed significantly longer median OS relative to those with *FOLH1*-Low tumors (median survival difference of 15.9 months in patients with biopsies derived from primary prostate [HR = 0.71, *P *< .0001], 11.6 months for lymph node metastases [HR = 0.67, *P *< .002], and 12.4 months for distant metastatic sites [HR = 0.61, *P *< .0001]) (Figure 5A-C), suggesting *FOLH1* is a positive prognostic factor. These results were also consistent for patients with liver and bladder metastases but not bone or lung (Figure 5D, Supplementary Figure S1). To begin to explore the predictive value of *FOLH1* expression in the entire adenocarcinoma prostate cancer cohort, we analyzed treatment outcomes for patients receiving lutetium ^177^Lu-PSMA-617 (*n *= 149). Among these patients, those with high *FOLH1* expression trended toward longer treatment-related OS (not reached in both groups; HR = 0.55; *P *= .16) (Figure 6A). Finally, we examined ARPI treatment-related OS in the entire prostate adenocarcinoma cohort, which also revealed improved OS for patients with high *FOLH1* expression (HR = 0.779, *P *< .001) (Figure 6B).

## Discussion

This comprehensive molecular analysis of a large cohort of prostate tumors provides new insight into molecular correlates and oncologic outcomes of *FOLH1*. First, our results demonstrate low *FOLH1* expression among tumors with neuroendocrine differentiation, as well as tumor site-specific differences in *FOLH1* expression. Second, our study revealed differential molecular and immune characteristics associated with *FOLH1* expression within specific tumor sites, and an association of *FOLH1* with AR signaling. Third, we demonstrate the prognostic value of *FOLH1* expression and its potential predictive value for ^177^Lu-PSMA-617 treatment. Collectively, these findings enhance our understanding of the role of *FOLH1*/PSMA in prostate cancer biology and its potential as a multifaceted molecular indicator to inform prognosis and therapeutic strategies in prostate cancer.

Among our findings, the relationship between *FOLH1* expression and tumor histology was particularly noteworthy. We observed significantly lower *FOLH1* expression in tumors exhibiting neuroendocrine differentiation compared to adenocarcinoma specimens, which was especially pronounced in pure high-grade neuroendocrine carcinoma. Even within the adenocarcinoma specimens, *FOLH1* expression displayed a significant, although modest, inverse correlation with the NEPC gene signature. These observations align with the known cellular localization of PSMA in AR-positive luminal epithelial cells, while basal cells and sporadic neuroendocrine cells in the epithelium are largely devoid of PSMA.^30–32^ Another study found that neuroendocrine differentiation led to suppression of the *FOLH1* gene,^33^ highlighting that tumors with neuroendocrine differentiation may have less favorable response to PSMA-targeted therapies.

Our analysis revealed significant site-specific variations in *FOLH1* expression, underscoring the presence of both inter- and intra-patient heterogeneity.^34^ Lymph node metastases exhibited the highest *FOLH1* expression levels, surpassing both primary prostate tumors and other metastatic sites. In contrast, visceral metastases, particularly those in the gastrointestinal tract, lung, and liver, showed significantly lower *FOLH1* expression. These findings align with recent studies on PSMA expression in prostate cancer. In IHC studies, moderate to strong PSMA staining has been reported in 91% (*N* = 52) of lymph node metastases.^35^ Conversely, Paschalis et al. observed lower PSMA expression in liver metastases using IHC, with only 35% of liver metastases (*N* = 40) showing high PSMA positivity.^7^ Sayar et al. also reported lower PSMA IHC H-scores in prostate liver metastases compared to other sites,^36^ lending further support to the hypothesis that the liver microenvironment is inherently capable of suppressing PSMA.^34^ While our study did not assess PSMA IHC expression, our results complement these existing data.

Our genomic analysis revealed distinct patterns of alterations associated with *FOLH1* expression levels across different tumor sites. A notable finding was fewer alterations in several tumor suppressor genes, including *PTEN* and *APC*, in *FOLH1*-High tumors, particularly in primary prostate sites. This pattern might indicate that tumors with high PSMA expression maintain a more differentiated state. Conversely, *FOLH1*-Low tumors, especially metastatic sites, exhibited increased mutations in genes in the Wnt and PI3K/AKT/mTOR pathways, the significance of which is multifaceted. The Wnt pathway plays a crucial role in stem cell maintenance and epithelial-to-mesenchymal transition, potentially contributing to a more aggressive and metastatic phenotype.^37^ Similarly, the PI3K/AKT/mTOR pathway is a key regulator of cell survival and proliferation, and its dysregulation has been linked to prostate cancer progression and therapy resistance.^38^ These findings align with previous studies suggesting that activation of these pathways may contribute to PSMA suppression and potentially to a more aggressive phenotype.^33^^,^^39^

Our analysis of the tumor immune microenvironment revealed a complex landscape in *FOLH1*-High tumors. A striking finding was the enrichment of M2 macrophages coupled with depletion of M1 macrophages in *FOLH1*-High tumors across all sites. This shift toward an M2-dominant phenotype is associated with tumor progression and immunosuppression in prostate cancer.^40^ Additionally, we observed significant depletion of CD8+ T cells in *FOLH1*-High tumors, suggesting an immune-cold environment. *FOLH1*-High tumors also showed enrichment of immune checkpoint gene expression, which are markers of T cell exhaustion and dysfunction.^41^ This pattern might indicate the presence of dysfunctional T cells within the depleted T cell population. Notably, *FOLH1*-High primary tumors exhibited higher rates of PD-L1 positivity, TMB-High status, and MMRd/MSI-High, features traditionally considered favorable for immunotherapy response. However, these characteristics were generally infrequent across the entire cohort and were not consistently observed in metastatic sites, highlighting the importance of considering tumor heterogeneity. The coexistence of immunosuppressive features (M2 macrophage dominance, reduced CD8+ T cells) with potential immunotherapy response markers (PD-L1 positivity, high TMB) in *FOLH1*-High tumors presents a complex scenario for treatment strategies and underscores the need for comprehensive immune profiling in these tumors. Ongoing clinical trials may provide crucial insights into how to effectively combine PSMA-targeted therapies with immunomodulatory approaches to address the immune environment in *FOLH1*-High tumors. These include the phase II study combining 177Lu-PSMA-617 with pembrolizumab (NCT03805594) and the NEPI trial (NCT06388369) testing neoadjuvant 177Lu-PSMA-617 with ADT and ipilimumab in high-risk patients.^42^ Another recent phase II study (NCT05150236) investigated the combination of 177Lu-PSMA-617 with ipilimumab plus nivolumab in patients with mCPRC. This combination resulted in prolonged PSA-PFS compared to 177Lu-PSMA-617 alone but was terminated early due to treatment-related myocarditis.^43^

Our study suggests that high *FOLH1* expression may be associated with improved OS in both primary and metastatic prostate cancer including liver and bladder metastases but not for patients with certain metastatic tumors such as bone and lung. Despite the limitations of the survival analyses and the potential for confounders, the consistency of this survival benefit across different disease sites, including liver, which is known to portend worse outcomes for patients with mCPRC,^44^ strengthens the case for *FOLH1* as a prognostic indicator in prostate cancer and may offer valuable insights for risk stratification and treatment planning. Regarding the predictive value of *FOLH1* expression for 177Lu-PSMA-617 therapy, our analysis revealed a trend toward longer treatment-related OS in patients with high *FOLH1* expression, although this did not reach statistical significance. This trend aligns with the biological rationale of PSMA-targeted therapies and suggests that *FOLH1* expression levels may help identify patients more likely to benefit from this treatment modality, although it is possible that this observation is a reflection of improved prognosis, independent of treatment. Well-controlled, prospective studies will be required to further investigate this hypothesis.

While these data are encouraging, inter- and intra-patient heterogeneity in *FOLH1* expression may influence treatment response and necessitates careful consideration in patient selection and treatment planning. Our findings complement those of the TheraP trial, which found that higher PSMA-PET SUV_mean_ was associated with greater PSA response and longer progression-free survival in patients receiving 177Lu-PSMA-617.^20^ Furthermore, recent analysis of the VISION trial data^16^ has led to the development a nomogram integrating PSMA SUV_max_, presence of PSMA-positive lymph nodes, and other clinical parameters to predict OS and progression-free survival in patients treated with 177Lu-PSMA-617.^45^ This nomogram demonstrates the potential for combining molecular and clinical factors to enhance patient selection and outcome prediction.

The dynamics of PSMA expression modulation by AR-targeted therapies, including the frequency, intensity, and timing of these changes, remain unclear.^46^ The potential synergistic or additive effects of combining PSMA-targeted therapy with AR-targeted therapy are still under investigation. For instance, the phase II Enza-P trial (NCT04419402) has reported enhanced PSA progression-free survival with the combination of 177Lu-PSMA-617 and enzalutamide compared to enzalutamide alone in men with risk factors for early progression on enzalutamide.^47^ Future analyses of serial imaging in such trials may provide further insights into how PSMA expression is modulated by AR inhibition and how this impacts treatment outcomes. Our findings underscore the need for further research to elucidate the mechanistic underpinnings of the PSMA-AR relationship. Consistent with results of a study by Weiner et al. that demonstrated longer cancer-specific survival for patients with *FOLH1*-High tumors treated with androgen deprivation therapy,^8^ our study found prolonged treatment-related OS for patients with *FOLH1*-High tumors treated with ARPI, underscoring the relationship between PSMA/*FOLH1* and AR signaling. It remains to be determined whether PSMA-targeted therapy and AR-targeted therapy can produce a consistent and significant synergistic effect through PSMA overexpression resulting from AR suppression, if they have an additive effect due to dual targeting of both PSMA and AR separately, or if AR suppression downregulates the target to negatively affect response to PSMA-targeted therapy.^16^^,^^48^ Furthermore, we noted a significant increase in *AR*-V7 splice site variants in *FOLH1*-High compared to *FOLH1*-Low primary tumors. Since AR-V7 contributes to ARPI resistance,^49^ one might expect that *FOLH1*-High tumors would exhibit greater ARPI resistance, in contrast to our findings showing longer ARPI treatment-related OS in patients with *FOLH1*-High tumors. However, this survival result must be interpreted with caution, as it may reflect the overall better prognosis of patients with *FOLH1*-High tumors. Specific studies to investigate the effects of *AR*-V7 splice variants in *FOLH1*-High tumors will be required, particularly given the complex mechanisms of full-length AR versus AR-V7 that mediate resistance to various ARPIs.^49^^,^^50^ Ongoing clinical trials, such as the PSMAddition trial (NCT04720157), which is testing the combination of AR signaling inhibitors with or without 177Lu-PSMA-617 in the metastatic hormone-sensitive prostate cancer setting, will provide valuable insights into the potential synergistic effects of these treatment approaches.

Limitations of this study include the use of claims data for survival analyses, which has inherent biases and does not allow for complete clinical annotation, including tumor stage, prior lines of therapy, performance status, or Gleason score. Thus, the survival analyses should be interpreted with caution, and additional prospective studies controlling for clinical variables will be required to validate our findings. Furthermore, while we stratified patients according to primary and metastatic tumor sites for outcome analyses in order to obtain a pure population of patients with metastatic disease, the primary tumor cohort likely includes patients with advanced/metastatic disease, since this only represents the site of biopsy and comprehensive molecular profiling remains more common in the metastatic setting. Finally, we were unable to characterize the relationship between *FOLH1* RNA and PSMA protein levels in our study. Most prior interrogations of PSMA have utilized IHC^4^^,^^7^^,^^51^ or PSMA-PET.^46^^,^^52^^,^^53^ Evaluation of *FOLH1* RNA expression as part of multi-panel gene testing for therapy guidance would make PSMA evaluation more accessible. Studies have established a strong correlation between PSMA protein and *FOLH1* RNA levels,^36^^,^^54^ and the rate of tumors with low *FOLH1* gene expression was also shown to be comparable to the rate of patients deemed ineligible for 177Lu-PSMA-617 according to PSMA radioligand uptake in the TheraP trial.^34^^,^^48^ Although a formal analysis of the correlation between PSMA radioligand uptake and *FOLH1* gene expression is needed, these studies support the use of gene expression as a proxy for assessment of PSMA protein.

## Conclusions

In this analysis of *FOLH1* gene expression in 7,082 prostate cancer specimens, we detected highly stratified expression between primary tumors and various metastatic tumor sites, with lymph node having the highest expression and liver having the lowest expression compared to primary sites. Differential genomics involving the Wnt and PI3K pathways were also observed according to *FOLH1* expression. Notably, *FOLH1*-High tumors had a lower M1:M2 ratio, reduced Treg and CD8+ T cells, elevated immune checkpoint expression and PD-L1 positivity, and an increased proportion of TMB-High tumors. High *FOLH1* expression positively correlated with AR signaling. Patients with metastatic tumors with high *FOLH1* expression also had improved prognosis and modestly improved benefit from ^177^Lu-PSMA-617. Given the expanding applications of PSMA-targeted therapies, including bispecific T-cell engagers and chimeric antigen receptor T-cell therapy,^55^ further investigation of the molecular correlates of *FOLH1*/PSMA could inform clinical trial design and application of such novel PSMA-targeted therapeutics in select patient populations.

## Supplementary Material

oyaf338_Supplementary_Data

## Data Availability

The deidentified sequencing data are owned by Caris Life Sciences and cannot be publicly shared due to the data usage agreement in place. These data will be made available upon request for replication and verification purposes.
