# Supplementary material for: Molecular and clinical correlates of high FOLH1 (PSMA) RNA expression in primary and metastatic prostate cancer
Source: Oncologist. 2025 Oct 7;30(11):oyaf338. doi: 10.1093/oncolo/oyaf338 (PMC12609168; doi:10.1093/oncolo/oyaf338)
Supplement: oyaf338_Supplementary_Data [file oyaf338_supplementary_data.docx]

**Supplementary Figure S1. Prognosis associated with *FOLH1* expression in metastatic sites of prostate cancer.** Kaplan-Meier curves show overall survival (OS), calculated from tissue collection to last contact, in *FOLH1*-High and *FOLH1*-Low groups among patients with bone metastases **(A)**, liver metastases **(B)**, bladder metastases **(C)**, and lung metastases **(D)**. *FOLH1* gene expression levels were stratified by quartiles 4 vs 1 (Q1:*FOLH1*-Low, Q4: *FOLH1*-High).

**
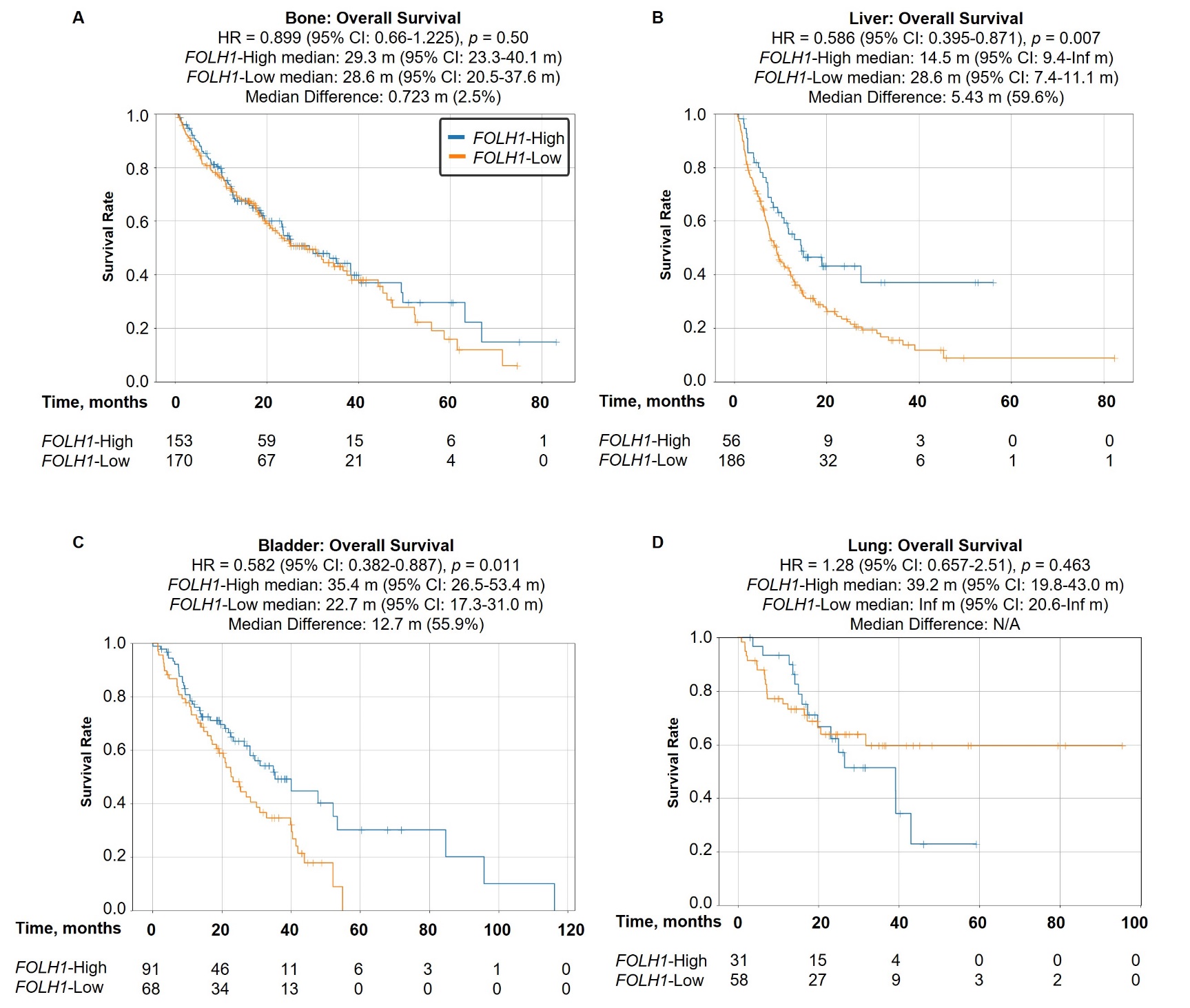
**
